# Isolation, characterization, and molecular identification of *Saccharomyces* strains from red dragon fruit peel (*Hylocereus polyrhizus*) for potential application in feed fermentation

**DOI:** 10.5455/javar.2026.m1013

**Published:** 2026-03-09

**Authors:** Nguyen Thi Hanh Chi, Tran Trung Tuan, Pham Duc Tho

**Affiliations:** 1 Department of Animal and Veterinary Sciences, Faculty of Agriculture and Natural Resources, An Giang University, An Giang, Vietnam; 2 Vietnam National University, Ho Chi Minh City, Vietnam

**Keywords:** *Saccharomyces cerevisiae*, red dragon fruit peel, yeast isolation, ITS sequencing, fermentation performance

## Abstract

**Objectives:** Red dragon fruit peel is a rich niche for indigenous yeasts with potential applications in feed fermentation. This study aimed to isolate, characterize, and molecularly identify *Saccharomyces* strains associated with red dragon fruit (*Hylocereus polyrhizus*) peel from An Giang province, Vietnam, and to assess their suitability as starter cultures for animal feed fermentation.

**Materials and Methods:** A total of thirty yeast strains were isolated, of which 11 exhibited growth on YPDA medium supplemented with sodium bisulfite (NaHSO₃), suggesting sulfite tolerance. Morphological and biochemical characteristics were assessed, and ITS-based DNA sequencing was performed for molecular identification.

**Results:** Six strains (CT6a, CT6b, CT6d, CT6g, CT3e, CT4a) showed high and stable biomass production and efficient fermentation performance in red dragon fruit peel extract. Sequence analysis confirmed all six as *Saccharomyces cerevisiae* with > 99% identity, and phylogenetic analysis further supported their clustering within the *S. cerevisiae* clade.

**Conclusions:** Red dragon fruit peel represents a promising substrate for fermentation, and the identified native *S. cerevisiae* strains demonstrate strong potential as starter cultures in biotechnological applications, particularly for animal feed fermentation.

## 1. Introduction

*Saccharomyces* is one of the most widely studied and utilized genera of yeasts due to its significant roles in both natural ecosystems and industrial applications. In nature, these yeasts contribute to nutrient cycling by decomposing organic matter, including plant and animal residues. Industrially, *Saccharomyces cerevisiae* is extensively employed in food and beverage production, particularly in baking, chocolate and coffee processing, and alcoholic fermentation, such as beer and wine. Its ability to enhance flavor, improve nutritional value, determine product quality, and prolong shelf life has been well documented [1]. Beyond food processing, *S. cerevisiae* also demonstrates considerable promise in animal nutrition. Its inclusion as a probiotic in livestock diets has been shown to reduce pathogenic bacteria, enhance feed conversion efficiency, and improve nutrient digestibility, ultimately mitigating the environmental impacts associated with livestock production [2]. Moreover, mannan oligosaccharides (MOS) derived from *S. cerevisiae* have been reported to increase beneficial gut microbes, such as *Lactobacillus* and *Bifidobacterium*, while suppressing pathogens, including *Salmonella, Escherichia coli, Clostridium perfringens*, and *Campylobacter*, in broiler chickens [3]. These findings suggest that yeast-based feed additives may offer a sustainable alternative to antibiotics in animal production, thereby contributing to the global effort to curb antimicrobial resistance. These roles emphasize the importance of isolating native *Saccharomyces* strains from fruit-based substrates.

Dragon fruit (*Hylocereus* spp.), a commercially important fruit widely cultivated in southern and south-central Vietnam, has seen rapid expansion in production. As of November 2024, Vietnam had approximately 50,000 hectares under dragon fruit cultivation, yielding around 1,119 thousand tons [4]. Notably, dragon fruit peels account for 22–44% of the total fruit weight [5], and are typically discarded during juice, bakery, and instant noodle production processes. The increasing volume of this agro-industrial waste poses environmental challenges if not effectively managed. One promising solution is the valorization of dragon fruit peels as animal feed. This not only reduces environmental burden but also contributes to cost savings in livestock production—an important consideration given the rising prices of commercial feeds. Solid-state fermentation (SSF) using microorganisms such as yeasts and molds is a widely recognized method for improving the nutritional value and palatability of agro-industrial by-products. Previous studies have demonstrated that SSF significantly improves protein content and nutrient availability in agro-industrial by-products [6, 21].

In this context, isolating and utilizing *Saccharomyces* strains capable of efficiently fermenting red-fleshed dragon fruit peels represents a strategic approach to simultaneously valorize agro-industrial waste and enhance the nutritional quality of animal feed. Such bioconversion processes are consistent with global priorities for sustainable agriculture, particularly in reducing antibiotic dependence and promoting animal health through probiotic-based interventions. Despite the recognized potential of *S. cerevisiae* in food and feed biotechnology, little information is available regarding the molecular identity and fermentation capacity of indigenous strains associated with red-fleshed dragon fruit peels in An Giang Province, Vietnam. Therefore, this study was designed to isolate, characterize, and molecularly identify native *Saccharomyces* strains from red-fleshed dragon fruit peels and to evaluate their potential application as starter cultures for feed fermentation.

## 2. Materials and Methods

### 2.1. Study period and location

This study was conducted over a 6-month period, from October 2024 to March 2025, at the Central Laboratory of An Giang University, Vietnam National University – Ho Chi Minh City.

### 2.2. Isolation of yeast strains from red-fleshed dragon fruit peel

Red-fleshed dragon fruits (*Hylocereus polyrhizus*) were purchased at a dragon fruit farm in An Giang. After peeling, the peels were cut into small fragments (0.3–0.5 cm), and 10 gm were transferred into 250 ml Erlenmeyer flasks containing 90 ml of YPD broth (10 gm/l yeast extract, 17 gm/l peptone, 20 gm/l glucose). The mixture was homogenized and incubated at 30°C for 24 h to enrich native yeast populations.

After incubation, cultures were serially diluted to 10^–1^ and 10^–2^, and 100 µl from each dilution was spread onto YPDA plates supplemented with 200 mg/l chloramphenicol to inhibit bacterial growth. YPDA was prepared by adding 17 gm/l agar to YPD broth. The selective medium used in both steps consisted of YPDA supplemented with 1 gm/l NaHSO₃ and 200 mg/l chloramphenicol to select for sulfite-tolerant yeasts, in line with previous reports on sulfite resistance in *S. cerevisiae* [16, 36]. Plates were incubated aerobically at 30°C for 24 h. Individual colonies with distinct morphology were selected and repeatedly sub-cultured to obtain pure isolates. Colony characteristics, including shape (circular or irregular), elevation (convex or flat), color (opaque white or translucent), and margin (entire or undulate), were recorded.

Preliminary identification was performed through microscopic observation of cell shape, budding patterns, and spore formation. Biochemical tests were used to assess the ability of isolates to ferment glucose, saccharose, and lactose, and to hydrolyze urea, following the classification [7, 8]. Biochemical assays included glucose/sucrose/lactose fermentation tubes (Merck, Germany) and urease hydrolysis using Christensen’s medium.

### 2.3. Screening of yeast strains with high fermentation capacity

To assess growth potential, single colonies were inoculated into 9 ml of Buffered Peptone Water (BPW, 25.5 gm/l), diluted, and adjusted to 10⁸ cells/ml using a Neubauer hemocytometer. Samples were serially diluted (10⁻¹–10⁻⁴) to obtain countable cell densities. 1 ml of the standardized suspension was transferred into a 50 ml Falcon tube containing 30 ml of YPD broth, and the mixture was incubated at 30°C with shaking at 200 rpm. At 24, 48, and 72 h, cultures were centrifuged at 5,000 rpm for 10 min, and wet biomass was weighed.

To evaluate fermentation performance, dragon fruit peels were blended with water at a 1:4 (w/v) ratio and hydrolyzed with 1% pectinase (Viscozyme^®^L, Novozymes, Denmark; activity 100 FBG/ml) at 50°C for 2 h. The mixture was filtered to remove solids. The extract was adjusted to 19°Brix with saccharose, pH was set to 4.0, and pasteurized with 140 mg/l sodium bisulfite (NaHSO₃) for 2 h. Cell viability was assessed using CFU enumeration on selective YPDA medium, following the procedure described by [12, 24] with minor modifications.

Yeast cultures were grown in YPD broth and standardized to 10⁸ cells/ml. Each fermentation assay included 9 ml of treated peel extract and 1 ml of yeast suspension in sterile test tubes, incubated at 30°C with shaking (200 rpm). At 24, 48, and 72 h, samples were diluted and plated onto YPDA supplemented with 200 mg/l chloramphenicol. CFU counts were recorded to monitor population dynamics. Strains with the highest biomass yield and viable counts were selected for molecular identification.

### 2.4. Molecular identification of selected yeast strains

Genomic DNA was extracted from selected yeast isolates. DNA extraction was performed using the Wizard^®^ Genomic DNA Purification Kit (Promega, Madison, WI, USA) according to the manufacturer’s instructions for yeast genomic DNA. The resulting DNA showed high purity (A_260_/A_280_ ≈ 1.8–2.0) and was suitable for downstream gene Internal Transcribed Spacer (ITS) amplification and sequencing. The gene ITS region was amplified via PCR using the primer pair ITS1 (5′-TCC GTA GGT GAA CCT GCG G-3′) and ITS4 (5′-TCC TCC GCT TAT TGA TAT GC-3′) according to [9]. PCR reactions (25 µl total volume) included 12.5 µl of 2x Master Mix, 1 µl of each primer (10 pmol/µl), 2 µl of DNA template, and 8.5 µl of DEPC-treated water. The thermal cycling protocol consisted of an initial denaturation at 94°C for 4 min, followed by 25 cycles of 94°C for 1 min, 48°C for 30 sec, 72°C for 1 min, and a final extension at 72°C for 10 min. PCR products were resolved on 1.5% agarose gels in 1x TAE buffer at 100 V for 35 min, stained with ethidium bromide (0.5 µg/ml), and visualized under UV illumination. A 100 bp DNA ladder was used as a reference marker, and amplicons of ~700–800 bp were considered positive. Five isolates showing single, strong bands of the expected size were selected for sequencing.

Amplicons were purified using a commercial PCR cleanup kit, and bidirectional Sanger sequencing was performed by a commercial provider (DNA Sequencing, Vietnam). Raw sequence chromatograms were quality-checked, trimmed, and assembled using BioEdit v7.2.5. Consensus sequences were aligned against reference sequences in NCBI GenBank using the BLASTn algorithm for species identification.

For phylogenetic analysis, ITS sequences from the selected strains were aligned with closely related sequences retrieved from GenBank using the ClustalW algorithm in MEGA X. A phylogenetic tree was constructed using the Neighbor-joining method and the Kimura 2-parameter model. The robustness of the tree was assessed with 1,000 bootstrap replications to determine confidence levels of the clades. The resulting tree was used to infer taxonomic and evolutionary relationships among the isolates.

### 2.5. Statistical analysis

All experimental data were tabulated and visualized using Microsoft Excel 2019. Statistical analysis was performed using the General Linear Model (GLM) procedure in Minitab version 16. One-way analysis of variance (ANOVA) was conducted to test for significant differences among treatments, and Tukey’s Honestly Significant Difference (HSD) test was applied for post hoc comparisons at a significance level of *p* < 0.05.

## 3. Results

### 3.1. Isolation and identification of Saccharomyces

#### 3.1.1. Isolation and morphological characterization

**Figure 1.**
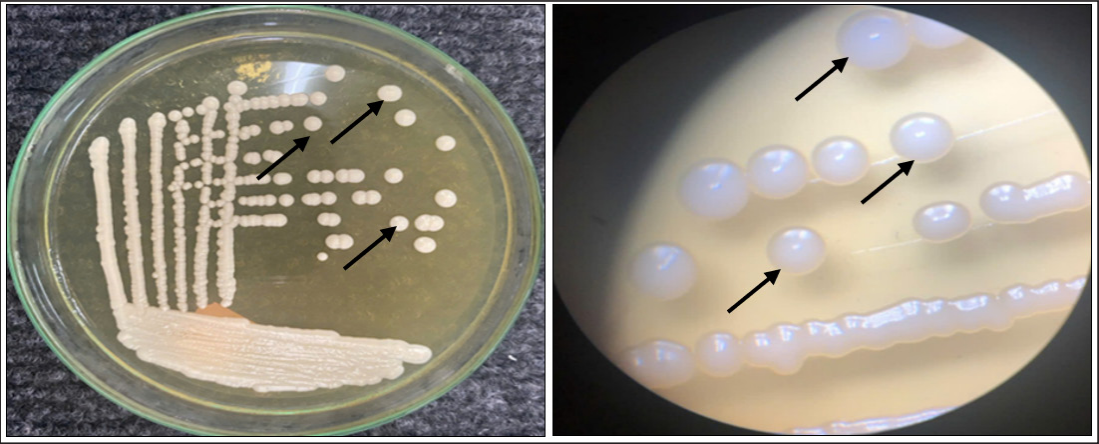
*Saccharomyces* colonies (black arrows) on YPDA medium and under a stereomicroscope.

**Figure 2.**
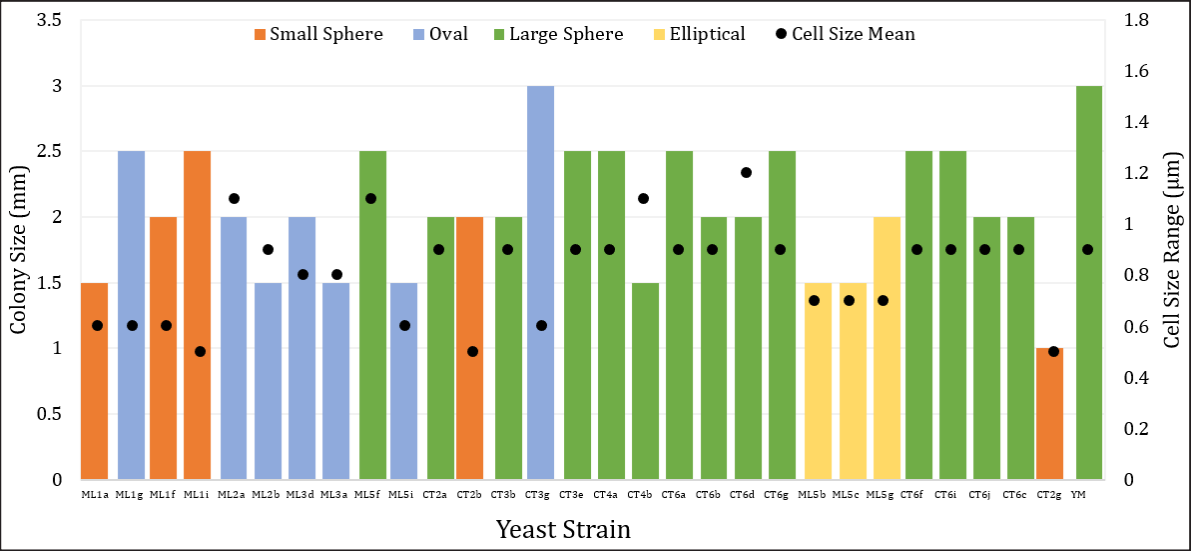
Morphological characteristics of yeast strains presumably belonging to the genus *Saccharomyces* isolated from red dragon fruit peel.

**Figure 3.**
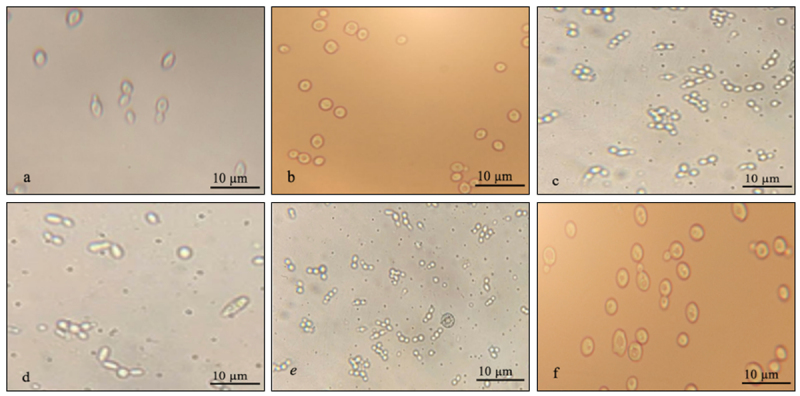
Cell morphology and budding patterns of yeast (objective lens x40). a – oval shape; b – large spherical shape; c – small spherical shape; d – elliptical shape; e – multipolar budding; f – unipolar budding.

A total of 30 yeast strains were successfully isolated from red dragon fruit peel *(Hylocereus polyrhizus*) collected in An Giang Province, Vietnam (Figures 1, 2). The isolates—coded YM, ML1a, ML1f, ML1i, ML1g, ML2a, ML2b, ML3d, ML3a, ML5b, ML5c, ML5g, ML5f, ML5i, CT2a, CT2b, CT2g, CT3b, CT3g, CT3e, CT4a, CT4b, CT6a, CT6b, CT6c, CT6d, CT6g, CT6i, CT6f, and CT6j—were preliminarily identified based on colony morphology and microscopic characteristics according to the classification [7, 8]. Most colonies appeared circular, with entire margins and milky-white to opaque pigmentation, ranging in diameter from 1.0–3.0 mm (Figure 3). Microscopy revealed diverse morphologies, including spherical, oval, and elliptical cells with unipolar or multipolar budding. Cell size was re-measured using a calibrated ocular micrometer, yielding corrected values of 3.2–7.8 µm, consistent with typical *S. cerevisiae* morphology [10].

#### 3.1.2. Biochemical differentiation and Saccharomyces identification

**Figure 4.**
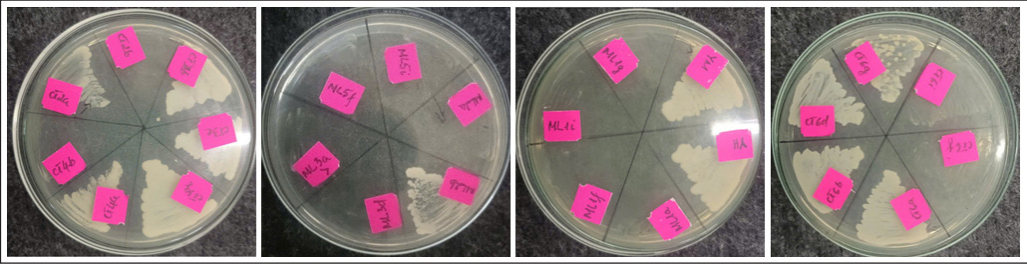
Yeast strains cultured on YPDA medium supplemented with 1 gm/l NaHSO₃ and 200 mg/l chloramphenicol.

Biochemical testing divided the 30 isolates into three distinct groups based on their fermentation of glucose, saccharose, and lactose, as well as urease activity (Table 1). The majority (24/30) fermented glucose and saccharose but not lactose and were urease-negative—traits characteristic of *Saccharomyces* species. Four strains fermented only glucose, suggesting affiliation with *Hanseniaspora*, while two fermented only saccharose, potentially belonging to Pichia or other non-*Saccharomyces* genera. Furthermore, 11 isolates (YM, CT2a, CT3b, CT3e, CT3g, CT4a, CT6a, CT6b, CT6d, CT6g, ML2b) demonstrated robust growth on selective YPDA medium containing 1 gm/l NaHSO₃ and 200 mg/l chloramphenicol—indicative of sulfite tolerance and supporting classification as putative *Saccharomyces* spp. (Figure 4).

**Table 1.** Results of sugar fermentation ability testing.

Yeast isolation	Sugar fermentation ability			Urease activity
	Glucose	Saccharose	Lactose	
YH (CT+), YM, ML1a, ML1f, ML1i, ML1g ML2a, ML2b, ML3d, ML3a, ML5f, ML5i, CT2a, CT2b, CT3b, CT3g, CT3e, CT4a, CT4b, CT6a, CT6b, CT6d, CT6g, CT6i, CT6j	+	+	–	–
ML5b, ML5c, ML5g, CT2g	+	–	–	–
CT6c, CT6f	–	+	–	–

**Note:**(+) indicates positive fermentation ability; (–) indicates no fermentation ability; CT+: control strain.

### 3.2. Evaluation of fermentation performance

**Figure 5.**
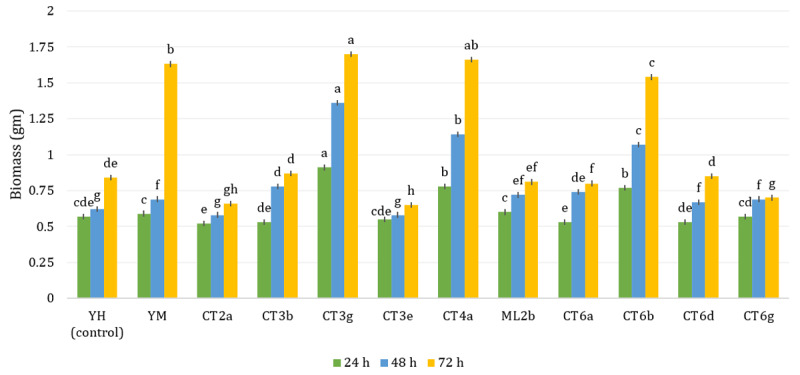
Biomass production of *Saccharomyces* strains after 24, 48, and 72 h of cultivation in red dragon fruit peel medium. Values are presented as mean ± SEM (*n* = 3). Different superscript letters above the bars indicate significant differences among strains within the same time point (*p* < 0.05; one-way ANOVA followed by Tukey’s HSD test).

The 11 isolates identified as putative *Saccharomyces* spp. were further assessed for fermentation efficiency by measuring biomass accumulation and viable cell density in red dragon fruit peel medium over 72 h. These parameters are critical indicators for selecting high-performance starter strains in fruit-based fermentations. As shown in Figure 5, strains CT3g, CT4a, and CT6b demonstrated superior biomass production at 24 h (0.91 gm, 0.78 gm, and 0.77 gm, respectively), outperforming the control (YH). Conversely, CT3b, CT6d, CT6a, and CT2a exhibited lower early-stage biomass, reflecting delayed adaptation. At 48 h- CT3g, CT4a, and CT6b maintained high productivity (1.36 gm, 1.14 gm, and 1.07 gm, respectively), while CT3b, CT6d, and CT6a showed accelerated growth. CT2a remained the least productive throughout. At 72 h- CT3g and CT4a reached peak biomass levels (1.70 gm and 1.67 gm), followed by CT6b (1.54 gm). Strains CT3g, CT4a, and CT6b exhibited significantly higher biomass production at all time points compared to the control (YH), with CT3g achieving the highest final biomass (1.70 gm). CT2a consistently produced the lowest biomass. The selected *Saccharomyces* isolates were further evaluated for fermentative potential in red dragon fruit peel extract over 72 h.

### 3.3. Growth dynamics in dragon fruit peel medium

Cell counts recorded during fermentation (Table 2) showed an exponential growth trend across all strains from 24 to 72 h, underscoring the suitability of red dragon fruit peel extract as a fermentation substrate. At 24 h, cell densities ranged from 2.00 × 10⁴ CFU/ml (CT3e) to 113 × 10⁴ CFU/ml (CT3g). The wide variation among strains reflects differences in lag-phase adaptation, metabolic efficiency, and initial inoculum viability. By 72 h, most strains exceeded 180 × 10⁴ CFU/ml, with CT6b reaching a maximum of 617 × 10⁴ CFU/ml. Among all isolates, CT6b exhibited the most consistent and robust growth across time points, with especially high cell densities at 24 h (75.7 × 10⁴ CFU/ml), 48 h (393 × 10⁴ CFU/ml), and 72 h (617 × 10⁴ CFU/ml). Other high-performing strains included CT6a, CT6g, CT6d, CT4a, and CT3e, all of which demonstrated sustained growth throughout the fermentation period.

**Table 2.** Yeast cell densities in Red Dragon fruit peel broth over propagation time (×10⁴ CFU/ml).

Treatment	Cell density at 24 h (CFU/ml)	Cell density at 48 h (CFU/ml)	Cell density at 72 h (CFU/ml)
YH (CT^+^)	78.7^b^	294^c^	475^e^
YM	56.3^c^	305^c^	415^g^
CT2a	3.00^ef^	148^de^	433^f^
CT3b	10.5^de^	324^c^	467^e^
CT3g	113^a^	164^de^	180^i^
CT3e	2.00^f^	135^e^	478^e^
CT4a	13.0^d^	172^d^	568^b^
ML2b	3.33^ef^	165^de^	245^h^
CT6a	62.2^c^	399^b^	475^e^
CT6b	75.7^b^	393^b^	617^a^
CT6d	57.7^c^	433^a^	510^d^
CT6g	83.8^b^	373^b^	548^c^
SEM	1.639	6.317	3.010
*p*	*p* < 0.001	*p* < 0.001	*p* < 0.001

**Note:** ^a, b, c, …^ Values within the same column followed by different superscript letters indicate statistically significant differences at *p* < 0.05; CT^+^: control strain.

### 3.4. Molecular identification and phylogenetic analysis

**Figure 6.**
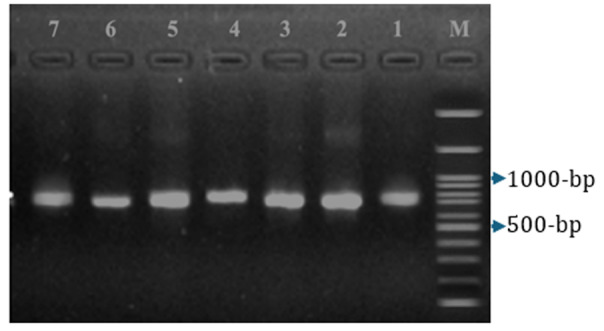
Agarose gel electrophoresis of PCR products for *S. cerevisiae* identification. Lane M: 100 bp DNA Marker; Lane 1: Positive control (YH); Lanes 2–7: CT6a, CT6b, CT6d, CT6g, CT3e, CT4a — all yielded positive amplification bands, indicating successful identification as *Saccharomyces cerevisiae*.

The molecular identity and taxonomic placement of the six yeast isolates (CT3e, CT4a, CT6a, CT6b, CT6d, and CT6g) were confirmed using PCR amplification and sequencing of the internal transcribed spacer (ITS) region—a widely accepted DNA barcode for fungal species identification (Figure 6). PCR amplification yielded single ~700–800 bp bands for all strains. BLASTn analysis confirmed > 99% identity with *S. cerevisiae*.

**Figure 7.**
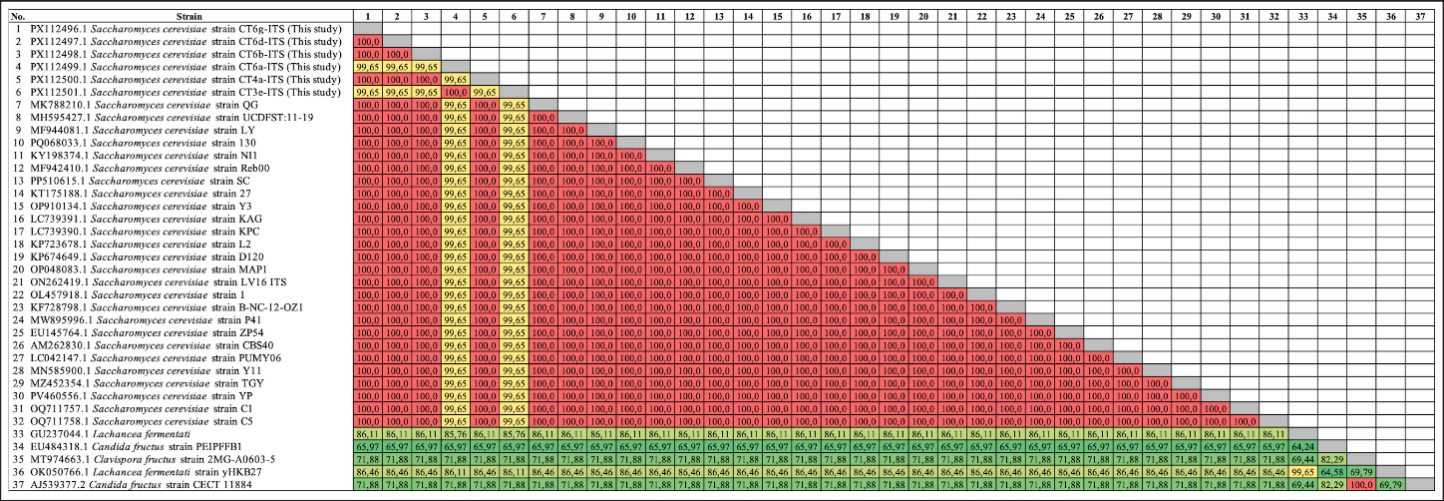
Pairwise genetic similarity matrix of yeast strains based on ITS sequences. The heatmap shows the percentage similarity between yeast isolates and reference strains. Red indicates high similarity (100%), yellow represents moderate similarity (> 99%), and green denotes low similarity (< 90%), corresponding to inter-species or inter-generic divergence. The six isolates from this study (CT3e, CT4a, CT6a, CT6b, CT6d, CT6g) exhibit > 99.65% identity among themselves, confirming their classification as *Saccharomyces cerevisiae*.

PCR was performed using universal ITS primers, and the resulting amplicons were sequenced and aligned against reference sequences from the NCBI GenBank database. The pairwise sequence similarity matrix (Figure 7) revealed extremely high identity values among the six isolates, ranging from 99.65% to 100%, indicating a high degree of genetic relatedness consistent with *S. cerevisiae*. Notably, three strains (CT6d, CT6b, and CT4a) showed complete sequence identity (100%), suggesting they may be genetically identical or derived from a common ancestor.

**Figure 8.**
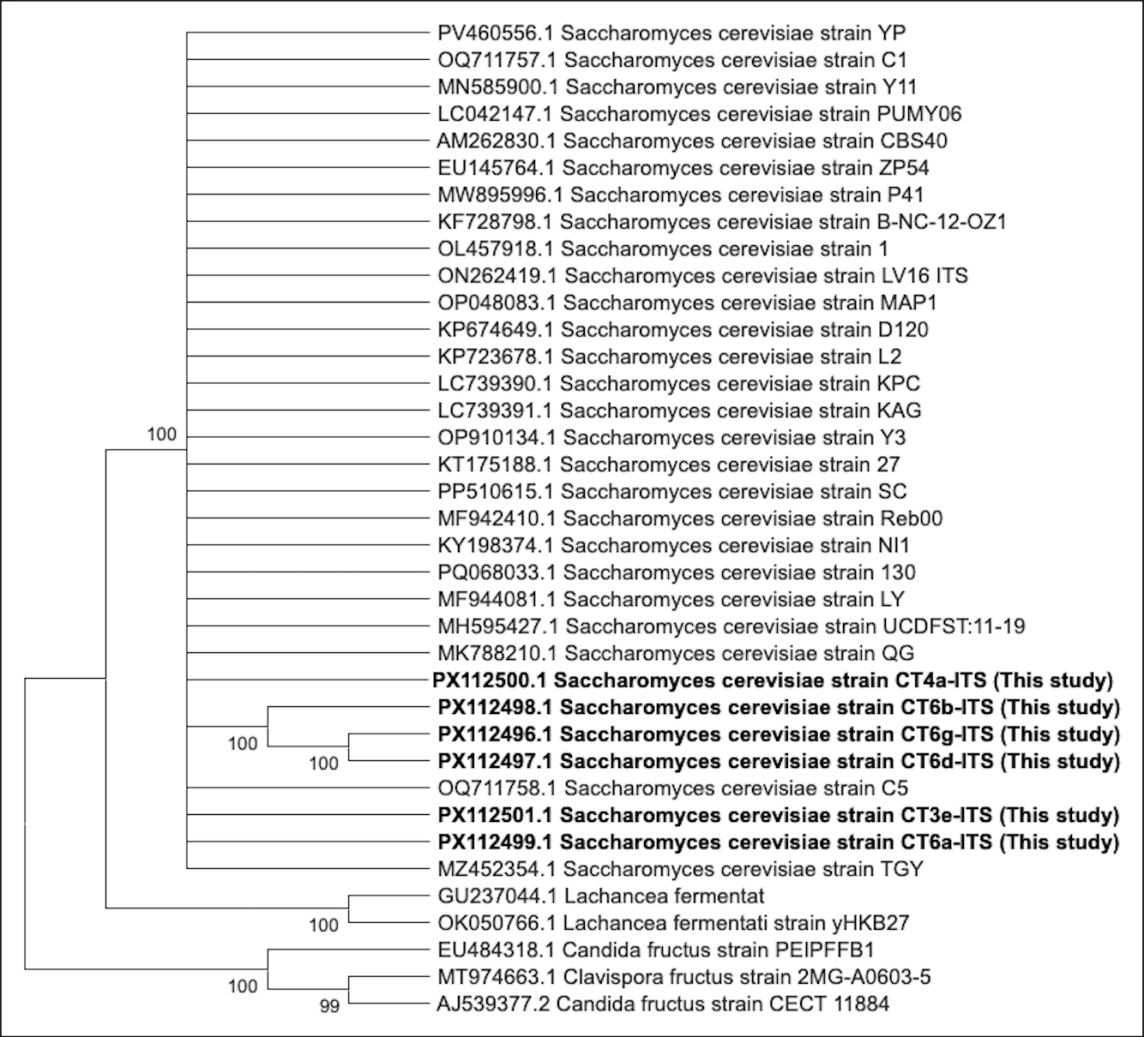
Phylogenetic tree based on ITS sequences of yeast isolates and reference strains. The Neighbor-Joining tree was constructed using MEGA X with 1,000 bootstrap replicates. Six isolates from this study (highlighted in bold) clustered within the *Saccharomyces cerevisiae* clade with strong bootstrap support (≥ 99%), confirming their taxonomic identity. Non-*Saccharomyces* species, such as *Lachancea, Candida*, and *Clavispora*, formed distinct outgroups, supporting the molecular resolution of ITS-based phylogenies.

These results were corroborated by phylogenetic analysis of ITS sequences. The Neighbor-Joining tree (Figure 8), constructed with 1,000 bootstrap replications, placed all six PCR-amplified isolates within the *S. cerevisiae* clade with strong bootstrap support (100%). Within this clade, CT4a, CT6b, CT6g, and CT6d formed a tightly grouped subcluster, while CT3e and CT6a appeared as a sister group—further reinforcing their classification as *S. cerevisiae*.

In contrast, non-*Saccharomyces* reference species—*Lachancea fermentati, Candida fructus*, and *Clavispora fructus*—clustered as distinct outgroups with high statistical confidence (bootstrap ≥ 99%) and showed markedly lower sequence similarity (42.44%–73.61%), as seen in the lower portion of the similarity matrix.

## 4. Discussion

The present study presents a comprehensive characterization of indigenous *Saccharomyces cerevisiae* strains isolated from red dragon fruit (*Hylocereus polyrhizus*) peel, incorporating morphological, biochemical, physiological, and molecular analyses. This integrative approach substantiates the potential applicability of these isolates in fermentation-driven processes, particularly within the domains of feed biotechnology and animal nutrition.

Morphologically, all isolates exhibited consistent colonial features—smooth-edged, milky-white colonies—while showing notable variability in cellular morphology, ranging from spherical to elliptical. These traits conform to classical taxonomic descriptors of *S. cerevisiae* [7, 8] and align with previously reported characteristics of fruit-derived yeast isolates [11, 12]. Micrometric analysis revealed cell sizes ranging from 3.2–7.8 µm, closely matching values reported in similar studies on fruit-origin yeasts [13, 14]. These dimensions are substantially smaller than those reported for *S. cerevisiae* strains isolated from sugar palm sap (3.8–10.5 µm) by Korabecna [9], suggesting that niche-specific factors, such as osmotic pressure, substrate complexity, and nutrient availability, may drive morphological adaptation. Indeed, such cell-size variability is often associated with altered budding dynamics and stress-responsive phenotypes [15], which may confer selective advantages in polyphenol- and sugar-rich fruit matrices.

Biochemical assays confirmed that most isolates ferment glucose and sucrose, lack urease activity, and do not utilize lactose, a biochemical fingerprint consistent with *S. cerevisiae*. The sulfite-tolerant phenotype observed among 11 isolates under NaHSO_3_ stress conditions (1 gm/l) corroborates their taxonomic identity and industrial potential. Sulfite resistance, linked to upregulation of the *SSU1* gene and increased antioxidant enzyme activity, is a vital attribute in feed fermentation and oenological contexts [16, 17].

In fermentation trials using red dragon fruit peel extract, selected strains (CT3g, CT4a, CT6b) exhibited superior biomass productivity and cell density, outperforming control strains and achieving peak values at 72 h. This performance is attributed to the metabolic versatility of *S. cerevisiae*, which can utilize hexose sugars and tolerate polyphenolic stressors [18, 19, 20]. The presence of betacyanins, flavonoids, and micronutrients in the peel enhanced respiratory efficiency and redox balance during exponential growth [21, 22].

From a microbial ecology standpoint, the consistent growth patterns across fermentation time points suggest that these strains are inherently compatible with complex plant-derived substrates. Comparative analyses reveal that the CFU levels attained here (up to 617 × 10⁴ CFU/ml) are on par or exceed those reported in studies utilizing optimized commercial media [23, 24, 25, 37]. These outcomes highlight the viability of low-cost agro-waste matrices for cultivating functional yeast biomass. Compared with other agricultural substrates such as cassava residue, pineapple waste, or soursop pulp, red dragon fruit peel supported equal or higher yeast biomass production, indicating its suitability as a competitive low-cost carbon source for feed fermentation [6, 11, 28].

The application of ITS-based sequencing allowed precise species-level resolution, confirming 100% similarity with reference *S. cerevisiae* strains. Phylogenetic reconstruction via the Neighbor-Joining method clustered all isolates into a monophyletic *S. cerevisiae* clade, with high bootstrap support (>99%) indicating genetic stability and taxonomic fidelity. This tight clustering suggests adaptive radiation of these strains in the sugar-rich peel niche, a phenomenon supported by recent reports on rapid substrate-driven domestication in *S. cerevisiae* populations [26, 27, 28].

In the context of veterinary microbiology, the indigenous *S. cerevisiae* strains identified in this study exhibit strong potential as direct-fed microbials (DFMs) for animal nutrition. Their high biomass productivity, sulfite resistance, and consistent fermentative performance align with the key safety and functionality benchmarks outlined by [36]. Recent studies have underscored the multifaceted benefits of DFMs, including enhanced ruminal fermentation efficiency, modulation of the host immune response, and reduced pathogenic microbial load in livestock production systems [23, 29, 30, 31]. Among the strains characterized, CT6b and CT4a, in particular, demonstrated phenotypic robustness and growth kinetics indicative of high probiotic potential. These findings warrant subsequent *in vivo* trials to assess their viability under gastrointestinal conditions, resistance to bile salts, and immunomodulatory capacity in ruminant or monogastric models.

While ITS analysis provides sufficient resolution for species identification, future studies should adopt whole-genome sequencing (WGS) and comparative transcriptomics to assess industrially relevant genes associated with thermotolerance, acid resistance, metabolite secretion, and biofilm regulation. Integrating metabolomic profiling under fermentation stress would enrich understanding of adaptive metabolic fluxes [32, 33]. Finally, valorizing red dragon fruit peel as a microbial reservoir contributes to circular bioeconomy strategies by converting agricultural by-products into valuable microbial inputs. This approach supports the conservation of local microbial biodiversity while simultaneously reducing feed production costs, an essential consideration under the growing environmental and economic pressures in livestock production systems. These objectives align closely with recent strategic directives from the FAO and the European Union that promote sustainable animal agriculture, protein security, and circular agri-food systems [34, 35].

## 5. Conclusions

This study successfully isolated and characterized *Saccharomyces* yeast strains from red dragon fruit peel (*H. polyrhizus*). Out of 30 isolates, six strains (CT6a, CT6b, CT6d, CT6g, CT3e, CT4a) exhibited strong biomass production and fermentation ability. Molecular identification confirmed these as *S. cerevisiae* with > 99% similarity. Their robust growth in fruit peel extract highlights their potential for application in feed fermentation and other agro-industrial bioprocesses.

## Data Availability

The data presented in this study are available from the corresponding author upon reasonable request.

## List of abbreviations

°Brix, degrees Brix; °C, degrees Celsius; bp, base pairs; cm, centimeters; gm, grams; gm/kg, grams per kilogram; gm/l, grams per liter; h, hours; mg/l, milligrams per liter; CFU/ml, colony-forming units per milliliter; ml, milliliters; µl, microliters; µm, micrometers; pmol/µl, picomoles per microliter; rpm, revolutions per minute; ANOVA, analysis of variance; A260/A280, absorbance ratio at 260 nm and 280 nm; BLASTn, Basic Local Alignment Search Tool for nucleotides; CFU, colony-forming unit; DNA, deoxyribonucleic acid; ITS, internal transcribed spacer; NCBI, National Center for Biotechnology Information; PCR, polymerase chain reaction; SEM, standard error of the mean; SSF, solid-state fermentation; TAE, Tris-acetate-EDTA; UV, ultraviolet; YPD, yeast peptone dextrose; YPDA, yeast peptone dextrose agar.
